# Toward Optimal Management of Behavioral and Psychological Symptoms of Dementia: Insights From a COVID-19 Pandemic Experience

**DOI:** 10.3389/fpsyt.2021.634398

**Published:** 2021-05-05

**Authors:** Karen Debas, Joanny Beauchamp, Christine Ouellet

**Affiliations:** Institut universitaire en santé mentale de Montréal, Integrated University Health and Social Services of the East-Island of Montreal, Montreal, QC, Canada

**Keywords:** COVID-19, dementia, major neurocognitive disorder, behavioral and psychologic symptoms of dementia, neuropsychiatric symptoms, non-pharmacological intervention, long-term care, caregiver

## Abstract

The first wave of SARS-CoV-2 has deeply affected long term care facilities in the province of Quebec. In response, governmental officials took protective measures, such as suspending visits and activities and even requiring residents to self-isolate to their room. Consequently, residents with major cognitive impairments were cut from their routine as well as from significant social interactions, support, and stimulation essential to their well-being. This isolation negatively affected many residents. For some of them, the loss of bearings resulted in newly or deteriorated behavioral and psychological symptoms of dementia (BPSD). These residents were then more at risk of contracting the virus or contaminating others. To face this challenge, hotels in the Greater Montreal area were transformed into temporary care facilities. As members of a multidisciplinary team specialized in the management of BPSD, we were asked to support the redeployed staff who had little experience in this domain. In this paper, we present the innovative tools implemented in this uncommon work setting. We also discuss factors identified as facilitating the care and treatment of people with BPSD. This experience leads us to propose avenues toward better BPSD management.

## Introduction

Behavioral and psychological symptoms of dementia (BPSD) are highly prevalent in patients with neurocognitive disorder (NCD), affecting more than 80% of patients in the course of their disease (1). BPSD includes a variety of heterogeneous symptoms, arising from interactions between dementia severity, environmental factors, unmet needs, somatic diseases, as well as personality and life experiences of the patient. According to a meta-analysis, the most frequent BPSD would be apathy (prevalence of 49%), followed by depression (42%), aggression (40%), anxiety (39%), sleep disorder (39%), irritability (36%), appetite disorder (34%), aberrant motor behavior (32%), delusion (31%), disinhibition (17%), and hallucination (16%) (2).

Other studies consider neuropsychiatric syndromes instead of single symptoms. According to one of them, neuropsychiatric symptoms could be classified into five distinct sub-syndromes: apathetic (as unique syndrome), affective (anxiety and depression), psychomotor (agitation, irritability, and aberrant motor behavior), psychotic (delusions and hallucinations), and manic (disinhibition and euphoria) (3).

It is important to address BPSD as it is associated with distress in nursing staff, special treatment needs as well as behavioral, medical and care complications (e.g., psychiatric consults, neurotropic drugs, physical/verbal aggressions, falls) (4).

Knowing the low efficacy of antipsychotic drugs concerning BPSD, and their deleterious side effects, there is now a consensus in the literature that non-pharmacological approaches should be the first choice of treatment when dealing with NCD related symptoms (5). These strategies encompass a large range of individually tailored interventions aimed at improving symptoms in patients and reducing caregiver stress by adapting routines, environment, or interaction with carers. However, implementing these approaches in real-world settings remains challenging for numerous reasons: poor access to expertise (6), unfamiliarity with non-pharmacological treatment among physicians, a well-established culture of psychotropic prescription to face “aggressive” behaviors (7, 8) and staff's lack of time and training (9).

Moreover, the COVID-19 pandemic brought its share of challenges regarding BPSD management. Since older adults were identified as a particularly vulnerable population group, Quebec authorities decreed restrictive measures for their protection. These measures were particularly drastic in long-term care facilities, where most residents suffer from major NCD. Residents were confined to their nursing homes and even to their room. Visits were banned and social activities were suspended, depriving residents of their usual interactions, stimulation, and routines. These protective measures forced residents to live in an impoverished and stressful environment, in which their basic psychosocial needs were not fulfilled. Studies have demonstrated the dramatic impact of quarantine on the clinical symptoms of patients with NCD. Some authors reported not only a worsening of cognitive function, but also an aggravation of behavioral symptoms in 51.9% of the patients, such as irritability, apathy, and agitation, or the onset of new behavioral symptoms for 25.9% of them, namely irritability, sleep disturbance, and agitation, with therapy adjustments required. Quarantine was also associated with an increase of caregiver's burden, mainly anxiety, depression, irritability, and distress (10).

Facilities for older adults in Quebec were particularly affected by the pandemic. The situation became critical in many of them. Medical care demands were high because of COVID infection among residents and many residences were understaffed because of the virus spreading among staff members. These conditions made residents' management difficult and propitious to adverse events. Also, contamination risks were hard to handle as many residents were not able to cope with the sanitary rules because of their cognitive state and limited adaptative mechanisms. Moreover, due to the disruptive impact of COVID-19 on the healthcare system, BPSD could not be addressed as needed. As seen in other countries (11), services dedicated to patients with NCD and BPSD were diminished, canceled or restricted to emergencies, decreasing the overall quality of care for these patients as well as impeding support and recommendations normally given to care facilities.

Since home care facilities were overwhelmed by the situation, some residents presenting BPSD were temporarily relocated to hotels of the Greater Montreal area. These were transformed into dedicated non-conventional sites, with the hope of limiting the risk of virus propagation.

In one of these hotels, members of our mobile team, composed of neuropsychologists and occupational therapists specialized in the management of BPSD, were dispatched to support the frontline health workers. Between April 8th and June 29th, 2020, one hundred and sixty-five residents were transferred to the hotel. To be admitted, the premise was that residents were not able to respect sanitary rules because of cognitive impairments. Only few exceptions included patients admitted based on the risk of contaminating another older adult living in the same space (e.g., spouse). Residents came from eastern Montreal, a multicultural district with lower economic conditions. They came from either retirement homes or intermediate care units. Except for the aforementioned particular cases, all residents presented major NCD with mild to moderate BPSD (wandering, depressive symptoms, repetitive questioning, following carers closely, refusal of collaboration for ADL). We estimate that over 90% of the residents required at least one intervention during their stay, while approximately half of them needed a more sustained implication of our team.

A few days after opening, many obstacles as to the care and security of people presenting NCD with neuropsychiatric symptoms were observed. Indeed, staff members deployed to hotels, such as nurses, physicians, and patient attendants, often came from different medical backgrounds. Most of the orderlies volunteering following a governmental appeal had no previous experience in the healthcare system at all. Finally, the physical environment was unfit for older people coping with the loss of independence: furniture and bathrooms were not adapted, corridors were dark and deprived of windows, emergency exits were easily accessible, and room doors could be locked from the inside. These features had the potential to generate more BPSD.

Working in this unconventional setting meant adapting our usual intervention methods. At least one member of our team was on-site Monday to Friday, from 6 A.M. to 10 P.M. We favored regular discussions directly with the staff to gather information about problematic situations, instead of the usual questionnaires or observational charts. We adapted our schedule to be able to exchange with staff members of every shift. To promote knowledge about BPSD, different teaching methods were used: direct feedback and coaching, modeling, discussions in small groups, and informative posters disposed on walls featuring notions of BPSD and strategies (refusal management, diversion, adapted active listening, etc.). Furthermore, to promote a person-centered approach, we wrote an information sheet of relevant information for most patients, with the help of a family member when possible. It was hung near the bedroom door, with the patient's or family's consent. Finally, we leveraged the technological advantage granted by WIFI access—which is lacking in most nursing homes –in the management of BPSD.

These new working conditions revealed many facilitators allowing to implement a person-centered approach and non-pharmacological strategies to address BPSD. One of the major takeaways from our experience was the speed with which staff members were able to learn the basic foundation of BPSD management, even with no prior experience in healthcare. Indeed, within a few weeks, we observed that regular staff members became more independent and seemed better at generating strategies by themselves while facing new behavioral challenges. In our opinion, this efficiency translated into benefits for the patients, who's distress seemed more easily soothed, daily care appeared to be less stressful and they seemed more quickly reassured when needed.

## Facilitators in the Management of BPSD

### Culture and Leadership

Realizing the challenges associated with BPSD, managers quickly required the full-time presence of our specialized team. Such inclusion among staff contrasted with our usual role of external consultants. The choice of the organization to integrate us emphasized the value of our presence from the beginning, it helped us to be perceived as allies and reinforced the importance of using our services when needed. Furthermore, they considered it essential to give employees access to the expertise required for the implementation of good practices. In different healthcare settings, leadership, and tangible support from the organization is known to be a key factor allowing to successfully apply a new skill (12, 13). It is also important to note that since we worked in a non-traditional site, this establishment had no organizational culture on which to build; no dominant mindset nor established protocol for the management of BPSD. Thus, staff members and managers seemed more willing to adapt to changes when necessary.

### Full-Time Presence

Working on-site brought many benefits. Staff quickly became familiar with our role and the type of situations in which we could be useful. Indeed, a home-made questionnaire administered at the closing of the hotel revealed that our regular presence brought a feeling of reassurance and relief for the majority of employees (88.2% agreed or strongly agreed with this statement). It also granted us unusual flexibility in providing opportunities for multiple learning exposures facilitating knowledge translation (14). We adapted our work to staff needs and occurring problematics and we delivered teaching according to staff availability to ensure receptivity. Also, we could modify our schedule to reach staff members from all shifts, which was essential knowing that some BPSD appear at specific times of the day or with specific caregivers. Having access to local experts dedicated to support staff has been demonstrated to be beneficial for patients in acute care settings (15).

Evidently, the ease of communication between our team and staff members accelerated the pace at which a problematic situation could be solved. We could intervene as soon as required, avoiding filling long requests and delays in care support. We were able to collect, directly from employees involved, the information pertaining to the challenging behavior, its circumstances of appearance, the approach used, and the subsequent reactions. Consequently, we benefitted from more detailed information than if gathered from a postponed debriefing or a third party. Finally, elaboration and adjustments of the intervention plans were faster than usually possible, allowing to solve a problematic dynamic before it crystallized.

### Teaching Methods

Our new work environment was conducive to the use of numerous teaching methods to promote knowledge and ensure skills acquisition as to care provided to older adults. Firstly, as we could witness the ongoing interactions between patients and staff, we could offer immediate feedback and coaching to support novice staff members to adjust their approach to reduce BPSD. Feedback was given during or immediately after the intervention, making it specific, and based on observations. Timely and concrete feedback is known to be a powerful tool to promote learning (16). Also, we could ourselves participate in care when necessary, which offered opportunities to demonstrate strategies and thus served as modeling experience. For instance, when observing a conversation between a patient asking for hairspray and an attendant refusing by providing lengthy explanations, we would gently slip into the conversation and reassure the patient saying we would take care of the situation. A comeback with the attendant was done to explain, as she observed, how her answer could be modified in order to adapt it to the patient's comprehension capabilities and avoid an escalade. Multimodal educational interventions such as modeling and mentorship are recognized to facilitate integration of new skills (14).

### Innovative Tools

Patient attendants and orderlies were the ones providing care and interacting with patients on a daily basis. However, they had no access to personal information about them, such as their past, habits, personality, and usual reactions. In addition, most patients could not properly communicate their needs because of their cognitive impairments. It is often the case that personal information is kept confidential, yet it complicates the individualized approach recommended for BPSD management. To overcome this issue, we wrote an information sheet, for patients presenting neuropsychiatric symptoms. It comprised information to facilitate interactions and behavior management, namely the patient's main interests, prior occupation, meaningful souvenirs, strategies recommended, and so on. Special attention was paid to avoid prejudicial or confidential data. This sheet was also meant to be collaborative, not only to demonstrate we valued staff's observations, regardless of their role or title, but also to facilitate communication between staff members, a challenge often reported in the management of BPSD (17). Having access to these personal facts was judged to be “essential” or “very useful” by nearly 95% of the staff surveyed to be efficient in their interventions. Our clinical impression is that the impact was most positive for patients, who could benefit from personalized care, meaningful conversations, and activities corresponding to interests.

Another new tool we integrated at the hotel was the support of technology to our interventions. Thanks to donations, one or two electronic tablets were provided on each floor (of 14–22 patients). We soon realized these devices were powerful avenues in managing and preventing BPSD. Possibilities were almost infinite, ranging from ludic applications, internet sites for music and videos, to different kinds of social media and video calls to maintain a connection with loved ones. The choice of application could be adapted to the person's interests, needs and capacities, sometimes with trial and error. We noticed benefits on patients' mood, collaboration and level of agitation when using electronic devices, especially when used to support activities (e.g., choral) or ADL (e.g., meals taken in video calls with a family member to stimulate appetite).

### Motivation

Unprecedented times has revealed a particular propensity to help in a proportion of the population. Orderlies who volunteered to work in a COVID-19 environment were generally touched by the situation of older adults depicted in the media and came with a motivation to serve and to contribute. Of course, the fact that their initial familiarity with cognitive impairments and BPSD was low could have boosted their curiosity and their inclination toward learning. Yet, their disposition evoked the concept of intrinsic motivation, which is known to allow greater receptivity, openness, and cognitive availability for new notions taught (18). Their attitude and eagerness to learn were remarkable. Furthermore, the fact that staff contributed to successful interventions may have increased motivation to integrate the proposed person-centered approach via basic reinforcement principles.

### The Perceived Role of Patient Attendants and Orderlies

The novelty of the environment allowed us to introduce an optimal model of BPSD management. We emphasized from the beginning the legitimacy of non-pharmacological interventions and we underlined that engaging in those interventions was as important as taking care of other needs, such as ADL. We taught staff members that encouraging social interactions was a powerful tool to prevent BPSD. Orderlies quickly integrated patients' occupation as a central part of their role and valued their engagement into residents' well-being, even though it wasn't explicitly part of their task description when hired. Study report that patient attendants are not encouraged to communicate small but important observations that could help in the management of BPSD (17). This is in contrast with the philosophy of care we put forward at the hotel. From our clinical perspective, the adoption of this more holistic vision brought more satisfaction for caregivers and deeper connections with patients, which also benefited the patients.

### Multidisciplinarity

We knew before the pandemic that disciplines such as ours are complementary in managing BPSD. Each profession has expertise specific to its field of interest, which is best suited for different situations. At the hotel, caseloads were naturally divided according to one's expertise, with transparent communication between professions (e.g., a neuropsychologist would lead cases where psychological symptoms were prominent, whereas an occupational therapist would be requested for cases of resistance during hygiene). The integration of psychosocial disciplines are essential parts of the equation in the success of BPSD management (19), and our experience persuaded us of the relevance of this type of expertise among staff members, who are used to a dominant medical model of care.

### Staff Ratio

One of the major contrasts in our work conditions, as opposed to those of the standard health care system, was the higher ratio of staff member/patient (orderlies and attendants combined). Indeed, the ratio at the hotel was on average 1 for 3.3 patients, as opposed to 1 for 5–7 patients in regular clinical settings in Quebec. Although the COVID environment meant that tasks took more time to assure safety and lower the contamination risks, staff availability was nevertheless more considerable than usual. More staff was present to address patients' needs and to do so in an individualized manner. They had more time to concentrate on a single patient if needed. Support from peers was easier to find in case of a complex situation. Also, staff members were more receptive when feedback was provided regarding a specific situation. Finally, stability of the staff was an important stake. Qualitatively, we noticed that the regular staff came to know each patient's particularities, which was advantageous to patients who could benefit of a more personalized and constant approach.

## Discussion

Numerous challenges pertain to the implementation of non-pharmacological interventions for BPSD. However, our recent experience as clinicians, in an atypical work setting brought by the COVID-19 pandemic, demonstrated it was possible for novice staff members to quickly integrate a person-centered approach with older adults having major NCD. The present article sheds light on multiple factors identified as contributing to the ease with which the staff acquired a new skill set to face mild to moderate BPSD. These facilitators refer to the culture and leadership, full-time presence of experts, diverse teaching methods, innovative tools, motivation, positive perceived role of staff, multidisciplinarity, and good staff ratio. We feel our observations are valuable to share in order to guide the scientific community, organizations, and clinicians, as to the factors we need to document and promote to better judge BPSD interventions (see Table 1 for facilitators and examples of their implications).

**Table 1:** Facilitators identified in the management of BPSD and concrete implications.

**Facilitators**	**Implications**
Culture and leadership	• Value the importance of using non-pharmacological strategies • Recognize the necessity to have access to experts
Full-time presence	• Have an expert working on site to ensure rapid contact • Have the expert dedicated to the task of supporting staff with BPSD management
Teaching methods	• Use knowledge translation science • Give timely and concrete feedback • Favor multimodal and multiple learning exposures
Innovative tools	• Be creative to favor access to relevant patient information and to improve communication among staff • Integrate technology into care
Motivation	• Develop motivation to facilitate learning of new skills • Facilitate successful interventions to increase motivation
Perceived role of patient attendants and orderlies	• Favor patient attendants and orderlies' contribution beyond activities of daily living • Involve them in the reflections and actions regarding BPSD management
Multidisciplinarity	• Integrate different disciplines to have a holistic perspective • Value psychosocial expertise
Staff ratio	• Acknowledge that a greater ratio allows more time to facilitate BPSD management

Until now, the facilitators allowing the implementation of non-pharmacological approaches have received little attention as compared to the approaches themselves. Although some interventions were shown to be efficient, a certain number of reviews report inconsistent results. The differences in the ways the interventions are defined, applied and measured, as well as the complexity to synthesize the studies, are suggested to explain these inconsistencies (8, 20–22). Yet, our experience had us question the importance given to a particular intervention, as opposed to the environment and conditions in which it is applied. Indeed, the facilitators identified above might account for a significant part of the variance when measuring the efficacy of an intervention.

Studying the effect of multi-level interventions is possible, but methodologically very challenging. The environment of care comprises many variables which can affect outcome measures, and trying to split elements of interventions are not thought to lead to beneficial outcome (13, 23). In our setting, we also noticed that the facilitators identified operated synergistically. For example, the high staff-patient ratio, and the fact that our team was easily accessible, played an important role to further help other facilitators to push through. The high ratio gave staff enough time to think about the challenging situations, request help when needed, and thus integrated more rapidly the strategies taught. It provided staff with the psychological and cognitive disposition sufficient to engage in demanding tasks, which is known to motivate a behavior change toward better practice (24). Also, the fact that we were entirely dedicated to supporting staff in the management of BPSD gave us the opportunity to use recommended teaching methods (14). As it happens, having access to direct feedback is one of three key elements suggested to facilitate the application of a skill, together with organizational culture and leadership (12). Moreover, the success of the application of a fostered practice highly depends on tangible support from managers (12, 13). A positive commitment of the organization attributes value to their employees, bringing them to perceive their role as pivotal for the well-being of the patient. This value translates notably into proper access to patient information, allowing personalized interventions. This type of management support sets conditions for staff members to feel proud, it improves job satisfaction and favors a stable workforce (13).

Our experience reiterated that optimal care for older adults with major NCD can only be achieved by adopting a holistic vision of care. Indeed, disturbing behaviors are often triggered by unfulfilled needs, arising when care is task-driven, as opposed to person-centered. Furthermore, relying on a dominant medical model of care is susceptible to lead to the neglect of psychosocial needs (25, 26). In that context, integrating various psychosocial disciplines is essential in order to succeed in the management of BPSD (19).

## Limitations

Due to the emergency situation of the pandemic, concepts presented in this paper are empirical and mostly rest on clinical observations. Indeed, data collection was hard to achieve for many reasons: patients were sent to the hotel with few information about their medical condition, our team was reoriented within <24-h notice preventing all scientific preparation, and it was not possible to prioritize data collection over our clinical role.

The environment described in this paper is an unconventional context, not representative of what is known from usual settings. Thus, the specific characteristics of the facilitators exposed are not meant to be transferable to all clinical contexts and cannot serve as formal recommendations. Instead, the perspective presented in this paper is rather an attempt to shed light as to the multitude of variables influencing the management of BPSD and its success.

## Conclusion

In conclusion, this paper highlights the vast number of variables implicated in the management of BPSD and the necessity to take them into account when measuring the efficacy of an intervention. To lean toward optimal care, future research would take advantage of looking beyond the interventions *per se*. Further analysis of the context in which the non-pharmacological intervention is applied could capture the complexity of BPSD and lead to greater clinical impact.

## Data Availability Statement

The raw data supporting the conclusions of this article will be made available by the authors, without undue reservation.

## Author Contributions

KD: worked full-time as neuropsychologist at the COVID-19 hotel. Substantial contribution to the conception and design of the work; literature search and appraisal of the quality of included papers; first draft of most parts of the work; acquisition, analysis, and interpretation of data; critical revision for important intellectual content; final approval of the version to be published; agreement to be accountable for all aspects of the work and ensure accuracy and integrity of all parts of the work. JB: worked full-time as occupational therapist at the COVID-19 hotel. Contribution to the conception and design of the work; first draft of some parts of the work; critical revision for intellectual content; final approval of the version to be published. CO: worked full-time as neuropsychologist at the COVID-19 hotel. Contribution to the conception and design of the work; literature search and appraisal of the quality of included papers; first draft of some parts of the work; critical revision for intellectual content; final approval of the version to be published. All authors contributed to the article and approved the submitted version.

## Conflict of Interest

The authors declare that the research was conducted in the absence of any commercial or financial relationships that could be construed as a potential conflict of interest.
